# THE IMPACT OF THE R3 PROGRAM ON HEALTH SERVICES UTILIZATION AND COSTS

**DOI:** 10.1093/geroni/igac059.823

**Published:** 2022-12-20

**Authors:** Jane Tavares, Elizabeth Simpson, Edward Miller, Pamela Nadash, Marc Cohen

**Affiliations:** University of Massachusetts Boston, Boston, Massachusetts, United States; University of Massachusetts Boston, Boston, Massachusetts, United States; University of Massachusetts Boston, Boston, Massachusetts, United States; University of Massachusetts Boston, Boston, Massachusetts, United States; University of Massachusetts Boston, Boston, Massachusetts, United States

## Abstract

Relatively few housing with services evaluations use rigorous designs when assessing program impacts on health services utilization. This study thus employed a pre/post difference-in-difference quasi-experimental design, including building-level comparisons using Medicare fee-for-service claims data across two stages of the R3 intervention. Intervention sites included seven buildings with roughly 1,200 individuals. Key outcomes included hospital admissions, 30-day hospital readmission, and emergency department admissions. Results indicate that adding the R3 intervention to low-income housing sites led to meaningful reductions in service utilization and costs when compared to buildings where the program was not operating. The introduction of risk-targeting in the second stage of the intervention further strengthened this effect. Findings demonstrate that residents of affordable senior housing communities, who tend to be in poorer health than their counterparts in the community, benefit from the R3 program and that additional investment in this type of initiative would benefit the health care system.

